# A systematic review and meta-analysis protocol examining the clinical characteristics and epidemiological features of olfactory dysfunction (OD) in coronavirus disease 2019 (COVID-19)

**DOI:** 10.1186/s13643-021-01624-6

**Published:** 2021-03-11

**Authors:** Rosemond Qian-Xiu Tan, Wai Tak Victor Li, Wing-Zi Shum, Sheung Chit Chu, Hang-Long Li, Yat-Fung Shea, Tom Wai-Hin Chung

**Affiliations:** 1 Li Ka Shing Faculty of Medicine, The University of Hong Kong, Hong Kong, China; 2 Division of Geriatrics, Department of Medicine, Li Ka Shing Faculty of Medicine, The University of Hong Kong, Queen Mary Hospital, Hong Kong, China; 3 Department of Microbiology, Li Ka Shing Faculty of Medicine, The University of Hong Kong, Queen Mary Hospital, Hong Kong, China

**Keywords:** COVID-19, SARS-CoV-2, Olfactory dysfunction, Anosmia, Smell loss, Systematic review, Meta-analysis, Protocol

## Abstract

**Background:**

The coronavirus disease 2019 (COVID-19) pandemic has caused recurring and major outbreaks in multiple human populations around the world. The plethora of clinical presentations of severe acute respiratory syndrome coronavirus 2 (SARS-CoV-2) has been described extensively, of which olfactory dysfunction (OD) was established as an important and common extrapulmonary manifestation of COVID-19 infection. The aim of this protocol is to conduct a systematic review and meta-analysis on peer-reviewed articles which described clinical data of OD in COVID-19 patients.

**Methods:**

This research protocol has been prospectively registered with the Prospective Register of Systematic Reviews (PROSPERO; CRD42020196202). CINAHL, ClinicalTrials.gov, Cochrane Central, EMBASE, MEDLINE and PubMed, as well as Chinese medical databases China National Knowledge Infrastructure (CNKI), VIP and WANFANG, will be searched using keywords including ‘COVID-19’, ‘coronavirus disease’, ‘2019-nCoV’, ‘SARS-CoV-2’, ‘novel coronavirus’, ‘anosmia’, ‘hyposmia’, ‘loss of smell’, and ‘olfactory dysfunction’. Systematic review and meta-analysis will be conducted according to the Preferred Reporting Items for Systematic Reviews and Meta-Analyses (PRISMA) and the Meta-analyses Of Observational Studies in Epidemiology (MOOSE) guidelines. Articles will be screened according to pre-specified inclusion and exclusion criteria to extract studies that include new clinical data investigating the effect of COVID-19 on olfactory dysfunction. Included articles will be reviewed in full; data including patient demographics, clinical characteristics of COVID-19-related OD, methods of olfactory assessment and relevant clinical outcomes will be extracted. Statistical analyses will be performed using the Comprehensive Meta-Analysis version 3.

**Discussion:**

This systematic review and meta-analysis protocol will aim to collate and synthesise all available clinical evidence regarding COVID-19-related OD as an important neurosensory dysfunction of COVID-19 infection. A comprehensive search strategy and screening process will be conducted to incorporate broad clinical data for robust statistical analyses and representation. The outcome of the systematic review and meta-analysis will aim to improve our understanding of the symptomatology and clinical characteristics of COVID-19-related OD and identify knowledge gaps in its disease process, which will guide future research in this specific neurosensory defect.

**Systematic review registration:**

PROSPERO registration number: CRD42020196202.

**Supplementary Information:**

The online version contains supplementary material available at 10.1186/s13643-021-01624-6.

## Background

The novel severe acute respiratory syndrome coronavirus 2 (SARS-CoV-2), the aetiological agent of the coronavirus disease 2019 (COVID-19) global pandemic, has infected over 102 million people worldwide, accounting for over 2,200,000 deaths as of 27 September 2020 [1]. Since its discovery in December 2019, the clinical signs and symptoms associated with COVID-19 infection have been described extensively by numerous research groups [2–5]. Consistent with the clinical characteristics of the 2003 SARS-CoV epidemic, the predominant clinical manifestations of COVID-19 affect the human respiratory system, ranging from silent hypoxia to respiratory failure and life-threatening acute respiratory distress syndrome (ARDS) [3, 6, 7]. In addition, COVID-19 was shown to be associated with olfactory dysfunction (OD), which has since been recognised as a common and important neurosensory impairment in COVID-19 [8–10].

Recent systematic reviews and meta-analyses regarding COVID-19-related OD have found significant discordance between subjective reporting of smell changes and objective quantitation of olfaction [11–13], suggesting that validated tools for the quantitative assessment of olfactory function, such as butanol threshold test (BTT) [14] and smell identification test (SIT) [15], may be more sensitive in identifying smell disturbances in COVID-19 patients. Interestingly, one article suggested that the prevalence of COVID-19-related OD was inversely related to the patients’ age, implying that young patients were more prone to experience smell disturbances in SARS-CoV-2 infection [12]. However, due to the limited scope of existing studies, the true global prevalence of COVID-19-related OD has not been accurately determined. More importantly, the duration and the long-term effects of COVID-19-related OD have not been adequately examined. Importantly, the potential associations of additional neurological deficits in COVID-19-related OD remain unknown.

In this systematic review and meta-analysis protocol, we aim to investigate the demographic characteristics of COVID-19 patients presenting with OD, and to ascertain whether there is any age, sex, or ethnic predisposition to COVID-19-related OD. In addition, we will investigate the potential associations between olfactory neurosensory impairments and other otolaryngologic or neurologic disorders in COVID-19 infection. Finally, we aim to determine the prevalence of COVID-19-related OD as an isolated symptom, including its onset and duration, and whether OD may be a prognostic indicator for COVID-19 disease severity.

## Methods/design

### Population

This systematic review will include peer-reviewed articles which described clinical data on OD in patients of all ages who were confirmed with SARS-CoV-2 infection by reverse transcription polymerase chain reaction (RT-PCR) tests.

### Study design

The systematic review protocol has been registered on the Prospective Register of Systematic Reviews (PROSPERO; CRD42020196202). The research progress will be periodically updated on PROSPERO. The systematic review and meta-analysis will be carried out according to the Preferred Reporting Items for Systematic Reviews and Meta-Analyses (PRISMA) [16] and Meta-analyses Of Observational Studies in Epidemiology (MOOSE) [17] guidelines (see Additional Files). The systematic review encompasses a qualitative review of case reports, case series, and observational studies for descriptive data analyses; followed by quantitative meta-analysis of the prevalence of COVID-19-related OD in order to explore the effect of OD and its relationship with neurological complications. The outcomes will be expressed as a coefficient using meta-regression [95% confidence interval (CI), *R*^2^ index and *p*-value] [18].

### Search strategy

For the systematic review, the research group will search CINAHL, ClinicalTrials.gov, Cochrane Central, EMBASE, MEDLINE and PubMed for articles published from 1^st^ January 2020 to the date of completion of data extraction. Search keywords include ‘COVID-19’, ‘coronavirus disease’, ‘2019-nCoV’, ‘SARS-CoV-2’, ‘novel coronavirus’, ‘anosmia’, ‘hyposmia’, ‘loss of smell’, and ‘olfactory dysfunction’. Additionally, articles published within this time period will be searched from the following Chinese medical databases: China National Knowledge Infrastructure (CNKI), VIP and WANFANG, to ensure greater scope of representation from different geographical and ethnic populations. The detailed search strings of each database can be found in Supplementary Table 1. To further increase the sensitivity of our search, the list of references from review articles relating to COVID-19-related OD will be screened manually to identify other potentially eligible articles. If data were missing or unclear, or we could not determine the nature of the outcome, we will contact the corresponding author of the publication by email for clarification.

Subsequently, the search results will be combined and duplicates will be removed by Excel (Microsoft Corporation, Washington, USA). Eligible articles will be screened by four authors (R.Q.X.T., W.T.V.L., W.Z.S. and S.C.C.) by the article titles and abstracts, followed by full text examination. Disagreements will be resolved by another author (T.W.H.C.).

### Study selection

**Fig. 1:**
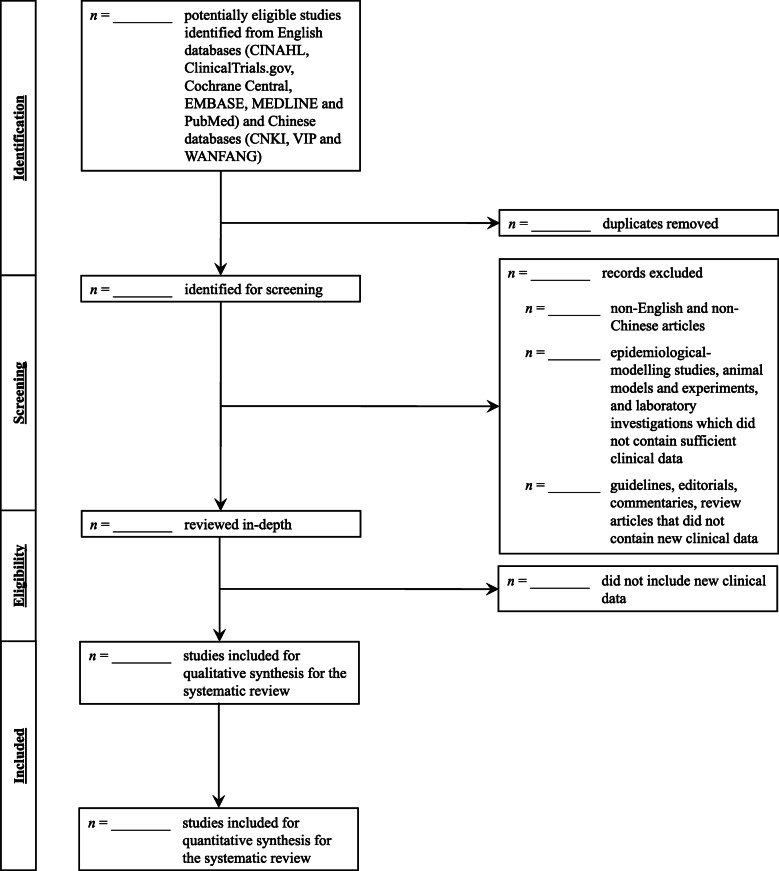
Preferred Reporting Items for Systematic Reviews and Meta-Analyses (PRISMA) flow diagram of the study protocol

Potentially eligible articles will be categorised using Microsoft Excel into three groups according to the article titles and abstracts: (A) articles containing clinical data on COVID-19; (B) epidemiological-modelling studies, animal models and experiments, and laboratory investigations which did not contain sufficient clinical data; and (C) guidelines, editorials, commentaries and review articles that did not contain new clinical data. After initial categorisation, full text of the articles containing clinical data [under category (A)] will be examined for their eligibility for inclusion. The design of the study selection strategy is summarised in Fig. 1.

The inclusion criteria for systematic review include (1) COVID-19 diagnosis confirmed by SARS-CoV-2 RT-PCR tests; (2) studies which reported clinical data on olfactory disturbances, either qualitatively or quantitatively; and (3) written in English or Chinese. The exclusion criteria include (1) articles which did not report individual clinical data on olfactory disturbances; and (2) articles that did not contain new clinical data. Case reports and case series of insufficient sample size (i.e. < 10 patients) will be included in the systematic review, but not the meta-analysis.

### Quality assessment

The methodological quality of studies will be determined using the Newcastle–Ottawa Scale (NOS) with a maximum of nine points (stars) for observational studies [19]. ‘Selection’, ‘Comparability’ and ‘Outcome’ will be the three categories included in the NOS for cohort studies. Selection (up to four stars) will include ‘representativeness of exposed cohort’ (i.e. COVID-19 patients reporting OD), ‘selection of non-exposed cohort’, ‘ascertainment of exposure’ [i.e. laboratory diagnosis of COVID-19 by RT-PCR; objective measurement of olfaction (e.g. BTT, SIT); and subjective reporting of olfactory disturbances] and ‘demonstration of outcome of interest was not present at the start of the study’ (i.e. elimination of patients with underlying medical conditions that may impair olfactory function). Comparability (up to two stars based on the design and analysis) will be defined as ‘comparison between COVID-19 patients with or without OD’. Outcome (up to three stars) will include ‘assessment of outcome’, ‘length of follow-up for outcomes to occur’ and ‘adequacy of follow-up of cohorts’. Nine stars are defined as the full score. Studies receiving 5–9 stars will be considered to be of high methodological quality, while articles rated 0–4 stars will be considered to be of poor methodological quality. Quality assessment will be independently confirmed for each of the included studies by two authors (T.W.H.C. and Y.F.S.) and disagreements will be resolved by consensus.

### Data Extraction

Data will be extracted independently by four authors (R.Q.X.T., W.T.V.L., W.Z.S. and S.C.C.). Disagreements will be resolved by mutual consensus. For included articles, the following data will be extracted: (1) basic information of the articles (first authors, country, and sample size); (2) patient demographics (age, sex and ethnicity); (3) disease characteristics [prevalence of abnormal olfaction, presence of associated otolaryngologic symptoms, presence of associated neurologic deficits, potential negative health outcomes (e.g. anorexia, skipped meals or weight loss), onset of OD relative to other symptoms of COVID-19, duration of COVID-19-related OD, overall clinical outcome]; (4) relevant investigation outcomes (e.g. viral load from the nasal or oropharyngeal cavity, relevant biopsy results); (5) the method(s) used to assess olfaction (qualitative or quantitative assessments, or both); (6) relevant imaging and endoscopic findings; and (7) any treatment provided.

### Statistical analysis

The prevalence of OD in COVID-19 patients will be computed for each of the studies. Pooled estimate of the prevalence of COVID-19-related OD will be calculated using the random effects meta-analysis, as the included studies involved different centres, different populations and different tools for olfactory assessment. Analysis of heterogeneity will be performed using the *I*^2^ statistics [20]. Publication bias will be evaluated by inspection of the funnel plot which will relate the standard errors of studies to their event rates. If inspection of the funnel plot suggested possibility of publication bias, the pooled prevalence of COVID-19-related OD will be corrected by calculation using the trim-and-fill method [21]. Egger’s test will also be performed [22]. The outcomes will be expressed as a coefficient, and this coefficient will be computed using meta-regression, with prevalence of OD as the dependent variable and the following covariates as independent variables. The measured covariates derived from included studies are sex ratio in the study, subject ethnicity (Caucasian, non-Caucasian), mean age of study subjects, presence of associated otolaryngology symptoms, presence of associated neurologic deficits, potential negative health outcomes, mean duration of COVID-19-related OD in days, proportion of subjects with COVID-19-related OD as the first symptom, and the mean SARS-CoV-2 viral load of relevant clinical specimens, the method(s) used to assess olfaction. The unit of proportion (%) shall be used for most of these variables. A *p* value less than 0.05 will be deemed statistically significant. All analyses will be performed using the Comprehensive Meta-Analysis version 3 (https://www.meta-analysis.com/index.php; Biostat, Englewood, NJ, USA). Descriptive statistics will be used for outcomes which are not suitable for meta-analyses.

## Discussion

This systematic review and meta-analysis will be the most up-to-date and comprehensive study that evaluates COVID-19-related OD. Meticulous search strategy will be applied to identify all relevant peer-reviewed articles from multiple medical databases, thereby increasing the sensitivity and specificity of the search strategy. One potential limitation of this meta-analysis will be the heavy reliance on observational studies, which may be prone to biases and confounding factors. However, quality assessment procedures as mentioned will help in the selection of articles. Strict adherence to the PRISMA and MOOSE guidelines will help to improve the reporting quality of the study. Additionally, this research protocol has been prospectively registered on PROSPERO, which aims to maintain transparency throughout the study process. Any amendments made in the process of the systematic review or meta-analysis will be clearly indicated on PROSPERO. The outcome of this systematic review and meta-analysis will be crucial in quantifying the global prevalence and disease burden of COVID-19-related OD and serve to identify knowledge gaps in understanding its disease course. This article will be instrumental for future research regarding this important neurosensory defect in the COVID-19 pandemic.

## Supplementary Information

**Additional file 1 **: **Table S1**. Search strings according to medical database platforms. PRISMA-P checklist. MOOSE checklist. PROSPERO registration.

## Data Availability

Not applicable.

## Abbreviations

ARDS: Acute respiratory distress syndrome
COVID-19: Coronavirus disease 2019
MOOSE: Meta-analyses Of Observational Studies in Epidemiology
NOS: Newcastle-Ottawa Scale
OD: Olfactory dysfunction
PRISMA: Preferred Reporting Items for Systematic Reviews and Meta-Analyses
PROSPERO: Prospective Register of Systematic Reviews
RT-PCR: Reverse transcription polymerase chain reaction
SARS-CoV-2: Severe acute respiratory syndrome coronavirus 2
