# Supplementary material for: A systematic review and meta-analysis protocol examining the clinical characteristics and epidemiological features of olfactory dysfunction (OD) in coronavirus disease 2019 (COVID-19)
Source: Syst Rev. 2021 Mar 11;10:73. doi: 10.1186/s13643-021-01624-6 (PMC7946443; doi:10.1186/s13643-021-01624-6)
Supplement: Supplementary file 1 — Additional file 1 : Table S1. Search strings according to medical database platforms. PRISMA-P checklist. MOOSE checklist. PROSPERO registration. [file 13643_2021_1624_MOESM1_ESM.zip › Additional_file_ pre-submissionR3.docx]

**ADDITIONAL FILES**

**Appendix:**

1. Supplementary table 1: Search strings according to medical database platforms
2. PRISMA-P checklist
3. MOOSE checklist
4. PROSPERO registration

**Supplementary table 1:** Search strings according to medical database platforms

| **Databases** | **Search strings** |
| --- | --- |
| **English medical databases** | |
| CINAHL | S1 TX COVID-19  S2 TX SARS-CoV-2  S3 TX 2019-nCoV  S4 TX Novel coronavirus  S5 TX Coronavirus disease  S6 TX Corona virus disease  S7 TX S1 or S2 or S3 or S4 or S5 or S6  S8 TX ((anosmi*) OR (hyposmi*) OR (microsmi*) OR (parosmi*) OR (dysomi*) OR (phantosmi*) OR (cacosmi*) OR (heterosmi*))  S9 TX ((olfactory OR olfaction) AND ((dysfunction*) OR (disturb*) OR (damage*) OR (abnormal*) OR (anomal*) OR (problem*) OR (issue*) OR (change*) OR (symptom*) OR (sign*) OR (malfunction*)))  S10 TX (((diminish*) OR (decreas*) OR (damp*) OR (reduc*) OR (loss of)) AND (smell sens*))  S11 TX (((sinonasal) OR (nasosinusal) OR (sinus) OR (nasal) OR (sinal) OR (turbina*)) AND ((dysfunction*) OR (disturb*) OR (damage*) OR (abnormal*) OR (anomal*) OR (problem*) OR (issue*) OR (change*) OR (symptom*) OR (sign*) OR (malfunction*)))  S12 TX S8 or S9 or S10 or S11  S13 S7 and S12  S14 S7 and S12, Limiter = Publication year 2020 |
| ClinicalTrials.gov | Condition or disease: COVID-19  Outcome measure: anosmia |
| Cochrane central | #1 ((2019 nCoV)) (word variations have been searched)  #2 COVID-19  #3 novel coronavirus  #4 coronavirus disease  #5 corona virus disease  #6 SARS-CoV-2  #7 #1 or #2 or #3 or #4 or #5 or #6  #8 ((anosmi*) OR (hyposmi*) OR (microsmi*) OR (parosmi*) OR (dysomi*) OR (phantosmi*) OR (cacosmi*) OR (heterosmi*))  #9 ((olfactory OR olfaction) AND ((dysfunction*) OR (disturb*) OR (damage*) OR (abnormal*) OR (anomal*) OR (problem*) OR (issue*) OR (change*) OR (symptom*) OR (sign*) OR (malfunction*)))  #10 (((diminish*) OR (decreas*) OR (damp*) OR (reduc*) OR (loss of)) AND (smell n2 sens*))  #11 (((sinonasal) OR (nasosinusal) OR (sinus) OR (nasal) OR (sinal) OR (turbina*)) AND ((dysfunction*) OR (disturb*) OR (damage*) OR (abnormal*) OR (anomal*) OR (problem*) OR (issue*) OR (change*) OR (symptom*) OR (sign*) OR (malfunction*)))  #12 #8 or #9 or #10 or #11  #13 #7 and #12 (with Cochrane Library publication date from Jan 2020 to Oct 2020) |
| EMBASE | 1. COVID-19.mp.  2. SARS-CoV-2.mp.  3. 2019-nCoV.mp.  4. novel coronavirus.mp.  5. coronavirus disease.mp.  6. corona virus disease.mp.  7. or/1-6  8. (anosmi* or hyposmi* or microsmi* or phantosmi* or cacosmi* or parosmi* or dysosmi* or heterosmi*).mp.  9. ((olfactory or olfaction) and (dysfunction* or disturb* or damage* or abnormal* or anomal* or problem* or issue* or change* or symptom* or sign* or malfunction*)).mp.  10. (diminish* or decreas* or damp* or reduc* or loss of).mp.  11. (smell adj2 sens*).mp.  12. 10 and 11  13. ((sinonasal or nasosinusal or sinus or nasal or sinal or turbina*) and (dysfunction* or disturb* or damage* or abnormal* or anomal* or problem* or issue* or change* or symptom* or sign* or malfunction*)).mp.  14. 8 or or 9 or 12 or 13  15. 7 and 14  16. limit 15 to yr="2020 -Current” |
| MEDLINE (Ovid) | 1. COVID-19.mp.  2. SARS-CoV-2.mp.  3. 2019-nCoV.mp.  4. novel coronavirus.mp.  5. coronavirus disease.mp.  6. corona virus disease.mp.  7. or/1-6  8. (anosmi* or hyposmi* or microsmi* or phantosmi* or cacosmi* or parosmi* or dysosmi* or heterosmi*).mp.  9. ((olfactory or olfaction) and (dysfunction* or disturb* or damage* or abnormal* or anomal* or problem* or issue* or change* or symptom* or sign* or malfunction*)).mp.  10. (diminish* or decreas* or damp* or reduc* or loss of).mp.  11. (smell adj2 sens*).mp.  12. 10 and 11  13. ((sinonasal or nasosinusal or sinus or nasal or sinal or turbina*) and (dysfunction* or disturb* or damage* or abnormal* or anomal* or problem* or issue* or change* or symptom* or sign* or malfunction*)).mp.  14. 8 or or 9 or 12 or 13  15. 7 and 14  16. limit 15 to yr="2020 -Current” |
| PubMed | (((COVID-19) OR (2019-nCoV) OR (coronavirus disease) OR (corona virus disease)) OR ((SARS-CoV-2) OR (novel coronavirus))) AND (((anosmi*) OR (hyposmi*) OR (microsmi*) OR (parosmi*) OR (dysomi*) OR (phantosmi*) OR (cacosmi*) OR (heterosmi*)) OR ((olfactory OR olfaction) AND ((dysfunction*) OR (disturb*) OR (damage*) OR (abnormal*) OR (anomal*) OR (problem*) OR (issue*) OR (change*) OR (symptom*) OR (sign*) OR (malfunction*))) OR (((diminish*) OR (decreas*) OR (damp*) OR (reduc*) OR (loss of)) AND (smell n2 sens*)) OR (((sinonasal) OR (nasosinusal) OR (sinus) OR (nasal) OR (sinal) OR (turbina*)) AND ((dysfunction*) OR (disturb*) OR (damage*) OR (abnormal*) OR (anomal*) OR (problem*) OR (issue*) OR (change*) OR (symptom*) OR (sign*) OR (malfunction*)))) AND (“2020/01/01”[Date - Publication] : “2020/09/11”[Date - Publication]) |
| **Chinese medical databases*** | |
| CNKI | #1 (Title, Keyword, Abstract) 新型冠状病毒肺炎 + 冠状病毒肺炎 + 新型冠状病毒  #2 (Full text) 新型冠状病毒肺炎 + 冠状病毒肺炎 + 新型冠状病毒  #3 (Title, Keyword, Abstract) COVID-19 + SARS-CoV-2 + 2019-nCoV + Novel coronavirus + Coronavirus disease + Corona virus disease  #4 (Full text) COVID-19 + SARS-CoV-2 + 2019-nCoV + Novel coronavirus + Coronavirus disease + Corona virus disease  #5 (Title, Keyword, Abstract) 嗅觉下降 + 嗅觉丧失 + 嗅觉减退 + 嗅觉障碍 + 嗅觉改变 + 鼻腔症状  #6 (Full text) 嗅觉下降 + 嗅觉丧失 + 嗅觉减退 + 嗅觉障碍 + 嗅觉改变 + 鼻腔症状  #7 (Title, Keyword, Abstract) Anosmia + Hyposmia + Microsmia + Phantosmia + Cacosmia + Parosmia + Dysomia + Heterosmia + Olfactory + Sense of smell + Sinonasal + Nasal + Nasosinusal + sinus  #8 (Full text) Anosmia + Hyposmia + Microsmia + Phantosmia + Cacosmia + Parosmia + Dysomia + Heterosmia + Olfactory + Sense of smell + Sinonasal + Nasal + Nasosinusal + sinus  #1 OR #2 OR #3 OR #4 AND #5 OR #6 OR #7 OR #8  Date: 1/1/20- 11/9/20 |
| VIP | #1 任意字段：2019新型冠状病毒 或  #2 任意字段：新型冠状病毒肺炎 或  #3 任意字段：新型冠状病毒 或  #4 任意字段：冠状病毒肺炎 与  #5 任意字段：嗅觉  全部期刊 |
| WANFANG | 检索表达式（中英文扩展&主题词扩展）： (((全部:("新型冠状病毒肺炎 + 冠状病毒肺炎 + 新型冠状病毒")+题名或关键词:(新型冠状病毒肺炎 + 冠状病毒肺炎 + 新型冠状病毒))*(全部:(嗅觉+嗅觉下降 + 嗅觉丧失 + 嗅觉减退 + 嗅觉障碍 + 嗅觉改变 + 鼻腔症状)+题名或关键词:(嗅觉+嗅觉下降 + 嗅觉丧失 + 嗅觉减退 + 嗅觉障碍 + 嗅觉改变 + 鼻腔症状))))*Date:2020- |

*Search strings for Chinese medical databases (CNKI, VIP, WANFANG) were formulated in simplified Chinese characters. CNKI: China National Knowledge Infrastructure.
