# Supplementary material for: A systematic review and meta-analysis protocol examining the clinical characteristics and epidemiological features of olfactory dysfunction (OD) in coronavirus disease 2019 (COVID-19)
Source: Syst Rev. 2021 Mar 11;10:73. doi: 10.1186/s13643-021-01624-6 (PMC7946443; doi:10.1186/s13643-021-01624-6)
Supplement: Supplementary file 1 — Additional file 1 : Table S1. Search strings according to medical database platforms. PRISMA-P checklist. MOOSE checklist. PROSPERO registration. [file 13643_2021_1624_MOESM1_ESM.zip › PROSPERO pdf copyR3.pdf]

National Institute for Health Research

UNIVERSITY of York
Centre for Reviews and Dissemination

Systematic review

1. * Review title.

Give the title of the review in English

A systematic review and meta-analysis protocol examining the clinical characteristics and epidemiological features of olfactory dysfunction in coronavirus disease 2019 (COVID-19)

2. Original language title.

For reviews in languages other than English, give the title in the original language. This will be displayed with the English language title.

3. * Anticipated or actual start date.

Give the date the systematic review started or is expected to start.

12/06/2020

4. * Anticipated completion date.

Give the date by which the review is expected to be completed.

30/09/2020

5. * Stage of review at time of this submission.

Tick the boxes to show which review tasks have been started and which have been completed. Update this field each time any amendments are made to a published record.
Reviews that have started data extraction (at the time of initial submission) are not eligible for inclusion in PROSPERO. If there is later evidence that incorrect status and/or completion date has been supplied, the published PROSPERO record will be marked as retracted.
This field uses answers to initial screening questions. It cannot be edited until after registration.

The review has not yet started: No

**Review stage**	**Started**	**Completed**
Preliminary searches	Yes	No
Piloting of the study selection process	Yes	No
Formal screening of search results against eligibility criteria	No	No
Data extraction	No	No
Risk of bias (quality) assessment	No	No
Data analysis	No	No

Provide any other relevant information about the stage of the review here.

6. * Named contact.

The named contact is the guarantor for the accuracy of the information in the register record. This may be any member of the review team.

Dr. Tom Wai-Hin Chung

Email salutation (e.g. "Dr Smith" or "Joanne") for correspondence:

Dr Chung

7. * Named contact email.

Give the electronic email address of the named contact.

tomchungwaihin@gmail.com

8. Named contact address

Give the full institutional/organisational postal address for the named contact.

9. Named contact phone number.

Give the telephone number for the named contact, including international dialling code.

10. * Organisational affiliation of the review.

Full title of the organisational affiliations for this review and website address if available. This field may be completed as 'None' if the review is not affiliated to any organisation.

Department of Microbiology, Li Ka Shing Faculty of Medicine, The University of Hong Kong, Hong Kong, China.

Organisation web address:

http://www.microbiology.hku.hk/

11. * Review team members and their organisational affiliations.

Give the personal details and the organisational affiliations of each member of the review team. Affiliation refers to groups or organisations to which review team members belong. **NOTE: email and country now MUST be entered for each person, unless you are amending a published record.**

Dr Tom Wai-Hin Chung. Department of Microbiology, Li Ka Shing Faculty of Medicine, The University of Hong Kong, Hong Kong, China.
Dr Yat-Fung Shea. Department of Medicine, Li Ka Shing Faculty of Medicine, The University of Hong Kong, Queen Mary Hospital, Hong Kong, China.
Rosemond Qian-Xiu Tan. Li Ka Shing Faculty of Medicine, The University of Hong Kong, Hong Kong, China.
Wai-Tak Victor Li. Li Ka Shing Faculty of Medicine, The University of Hong Kong, Hong Kong, China.
Sheung-Chit Chu. Li Ka Shing Faculty of Medicine, The University of Hong Kong, Hong Kong, China.
Wing-Zi Shum. Li Ka Shing Faculty of Medicine, The University of Hong Kong, Hong Kong, China.
Hang-Long Li. Li Ka Shing Faculty of Medicine, The University of Hong Kong, Hong Kong, China.
Dr Fergus Kai-Chuen Wong. Department of Ear, Nose and Throat Surgery, Pamela Youde Nethersole

Eastern Hospital, Hong Kong, China.
Dr Siddharth Sridhar. Department of Microbiology, Li Ka Shing Faculty of Medicine, The University of Hong Kong, Hong Kong, China.
Dr Vincent Chi-Chung Cheng. Department of Microbiology, Li Ka Shing Faculty of Medicine, The University of Hong Kong, Hong Kong, China.
Professor Ivan Fan-Ngai Hung. Department of Medicine, Li Ka Shing Faculty of Medicine, The University of Hong Kong, Hong Kong, China.
Professor Kwok-Yung Yuen. Department of Microbiology, Li Ka Shing Faculty of Medicine, The University of Hong Kong, Hong Kong, China.

12. * Funding sources/sponsors.

Details of the individuals, organizations, groups, companies or other legal entities who have funded or sponsored the review.

Shaw Foundation Hong Kong

Michael Seak-Kan Tong

Richard Yu and Carol Yu

May Tam Mak Mei Yin

Jessie & George Ho Charitable Foundation

Perfect Shape Medical Limited

Respiratory Viral Research Foundation

Hui Ming, Hui Hoy and Chow Sin Lan Charity Fund Limited

Sanming Project of Medicine in Shenzhen, China (SZSM201911014)

High Level-Hospital Program, Health Commission of Guangdong Province, China

Consultancy Service for Enhancing Laboratory Surveillance of Emerging Infectious Diseases and Research

Capability on Antimicrobial Resistance for the Department of Health of the Hong Kong Special Administrative

Region Government, Hong Kong, China

Theme-Based Research Scheme of the Research Grants Council of the Hong Kong Special Administrative

Region Government, Hong Kong, China

Grant number(s)

State the funder, grant or award number and the date of award

Sanming Project of Medicine in Shenzhen, China (SZSM201911014)

Theme-Based Research Scheme (T11/707/15) of the Research Grants Council of the Hong Kong Special

Administrative Region Government, Hong Kong, China

13. * Conflicts of interest.

List actual or perceived conflicts of interest (financial or academic).

None

The authors declare no conflict of interest

14. Collaborators.

Give the name and affiliation of any individuals or organisations who are working on the review but who are not listed as review team members. **NOTE: email and country must be completed for each person, unless you are amending a published record.**

15. * Review question.

State the review question(s) clearly and precisely. It may be appropriate to break very broad questions down into a series of related more specific questions. Questions may be framed or refined using PI(E)COS or similar where relevant.

[A] What are the clinical characteristics of olfactory dysfunction (OD) in coronavirus disease (COVID-19) patients infected by severe acute respiratory syndrome coronavirus 2 (SARS-CoV-2)?

1. What is the prevalence of isolated OD as the only symptom in COVID-19 patients?

2. What is the onset of OD relative to other symptoms of COVID-19 infection (i.e. OD as the first and presenting symptom of COVID-19)?

3. Is there an association between COVID-19-related OD and SARS-CoV-2 viral load?

4. What is the symptom duration of COVID-19-related OD?

5. What are the otolaryngologic symptoms associated with OD in COVID-19 infection, if any?

6. What are the neurologic symptoms associated with OD in COVID-19 infection, if any?

7. What are the health and lifestyle disturbances (e.g. skipped meals, loss of appetite, weight loss) associated with COVID-19-related OD?

8. Are there any risk factors that may predispose COVID-19 patients to develop OD?

9. What is the prognostic significance of COVID-19-related OD in the overall clinical outcome of SARS-CoV-2 infected patients, if any?

[B] What are the global epidemiological features of olfactory dysfunction (OD) in COVID-19 patients?

1. Are young COVID-19 patients more prone to OD?

2. Is there a gender difference in the prevalence of OD?

3. Is there an ethnic difference in the prevalence of OD?

16. * Searches.

State the sources that will be searched (e.g. Medline). Give the search dates, and any restrictions (e.g. language or publication date). Do NOT enter the full search strategy (it may be provided as a link or attachment below.)

PubMed, MEDLINE, EMBASE, Cochrane Central, ClinicalTrials.gov and CINAHL will be searched for articles published from 1st January 2020 to 11th September 2020. Search keywords include "COVID-19", "coronavirus disease", "2019-nCoV", "SARS-CoV-2", "novel coronavirus", "anosmia", "hyposmia" and "olfactory dysfunction". Additionally, searches for articles published in the same time period will also be conducted on the following major Chinese medical databases: China National Knowledge Infrastructure (CNKI), VIP and WANFANG, to ensure greater scope of representation. If the database does not allow for exact date searching, all articles within the year 2020 will be searched. English and Chinese articles will be included

17. URL to search strategy.

Upload a file with your search strategy, or an example of a search strategy for a specific database, (including

the keywords) in pdf or word format. In doing so you are consenting to the file being made publicly accessible. Or provide a URL or link to the strategy. Do NOT provide links to your search **results**.

https://www.crd.york.ac.uk/PROSPEROFILES/196202_STRATEGY_20200908.pdf

Alternatively, upload your search strategy to CRD in pdf format. Please note that by doing so you are consenting to the file being made publicly accessible.

Do not make this file publicly available until the review is complete

18. * Condition or domain being studied.

Give a short description of the disease, condition or healthcare domain being studied in your systematic review.

Olfactory dysfunction (OD) has been shown to be an important and common symptom in coronavirus disease 2019 (COVID-19). This systematic review and meta-analysis aims to collate and synthesise all clinical studies that meet prespecified eligibility criteria, in order to provide an in-depth description of the clinical characteristics of COVID-19-related OD, and elucidate the true global prevalence and epidemiological features of COVID-19-related OD.

19. * Participants/population.

Specify the participants or populations being studied in the review. The preferred format includes details of both inclusion and exclusion criteria.

Inclusion criteria: All patients with SARS-CoV-2 infection confirmed by reverse transcription polymerase chain reaction (RT-PCR)

Exclusion criteria: All patients without SARS-CoV-2 infection confirmed by reverse transcription polymerase chain reaction (RT-PCR)

20. * Intervention(s), exposure(s).

Give full and clear descriptions or definitions of the interventions or the exposures to be reviewed. The preferred format includes details of both inclusion and exclusion criteria.

Qualitative or quantitative documentation of smell impairment in laboratory confirmed COVID-19 patients

21. * Comparator(s)/control.

Where relevant, give details of the alternatives against which the intervention/exposure will be compared (e.g. another intervention or a non-exposed control group). The preferred format includes details of both inclusion and exclusion criteria.

Comparator: RT-PCR positive COVID-19 patients with olfactory dysfunction

Control: RT-PCR negative COVID-19 patients

22. * Types of study to be included.

Give details of the study designs (e.g. RCT) that are eligible for inclusion in the review. The preferred format includes both inclusion and exclusion criteria. If there are no restrictions on the types of study, this should be stated.

Inclusion criteria: all studies that report clinical data on olfactory dysfunctions in patients will be included, regardless of study type

Exclusion criteria: Literature reviews, comments, correspondences and letters to the editor which do not contain new clinical data will be excluded

23. Context.

Give summary details of the setting or other relevant characteristics, which help define the inclusion or exclusion criteria.

24. * Main outcome(s).

Give the pre-specified main (most important) outcomes of the review, including details of how the outcome is defined and measured and when these measurement are made, if these are part of the review inclusion criteria.

1. To investigate the global prevalence of olfactory dysfunction (OD) in COVID-19.
2. To investigate the clinical presentation of olfactory dysfunction (OD) in COVID-19.

[A] Olfactory dysfunction in COVID-19 patients, documented by qualitative assessments (e.g. self-reporting, surveys, questionnaires) only

[B] Olfactory dysfunction in COVID-19 patients, documented by quantitative measurements (e.g. butanol threshold test, smell identification test...)

This is achieved by only including articles that report clinical data on OD qualitatively or quantitatively.

* Measures of effect

Please specify the effect measure(s) for you main outcome(s) e.g. relative risks, odds ratios, risk difference, and/or 'number needed to treat.

Odds ratio (OR)

25. * Additional outcome(s).

List the pre-specified additional outcomes of the review, with a similar level of detail to that required for main outcomes. Where there are no additional outcomes please state ‘None’ or ‘Not applicable’ as appropriate to the review

To investigate if there is an age, sex, or ethnic predisposition to COVID-19-related OD, the potential association between olfactory neurosensory impairments and other otolaryngologic or neurologic disorders in COVID-19 infection, the prevalence of COVID-19-related OD as an isolated symptom, the onset and duration of COVID-19-related OD, and whether OD is a prognostic indicator for COVID-19 disease severity.

* Measures of effect

Please specify the effect measure(s) for you additional outcome(s) e.g. relative risks, odds ratios, risk difference, and/or 'number needed to treat.

Odds ratio (OR)

26. * Data extraction (selection and coding).

Describe how studies will be selected for inclusion. State what data will be extracted or obtained. State how this will be done and recorded.

Data will be extracted independently by four authors (R.Q.X.T., W.T.V.L., W.Z.S. and S.C.C.). Disagreements will be resolved by mutual consensus. For included articles, the following data will be extracted: (1) basic information of the articles (first authors, country, and sample size); (2) patient demographics (age, sex, and ethnicity); (3) disease characteristics [prevalence of abnormal olfaction, presence of associated otolaryngologic symptoms, presence of associated neurologic deficits, potential negative health outcomes (e.g. anorexia, skipped meals, or weight loss), onset of OD relative to other symptoms of COVID-19, duration of COVID-19-related OD, overall clinical outcome]; (4) relevant investigation outcomes (e.g. viral load from the nasal or oropharyngeal cavity, relevant biopsy results); (5) the method(s) used to assess olfaction (qualitative or quantitative assessments, or both); (6) relevant imaging and endoscopic findings; and (7) any treatment provided.

27. * Risk of bias (quality) assessment.

State which characteristics of the studies will be assessed and/or any formal risk of bias/quality assessment tools that will be used.

The methodological quality of studies will be determined using the Newcastle–Ottawa Scale (NOS) with a maximum of nine points (stars) for observational studies. ‘Selection’, ‘Comparability’ and ‘Outcome’ will be the three categories included in the NOS for cohort studies.

28. * Strategy for data synthesis.

Describe the methods you plan to use to synthesise data. This **must not be generic text** but should be **specific to your review** and describe how the proposed approach will be applied to your data. If meta-analysis is planned, describe the models to be used, methods to explore statistical heterogeneity, and software package to be used.

The prevalence of OD in COVID-19 patients will be computed for each of the studies. Pooled estimate of the prevalence of COVID-19-related OD will be calculated using the random-effects meta-analysis, as the included studies involved different centres, different populations and different tools for olfactory assessment. Analysis of heterogeneity will be performed using the I^2 statistics. Publication bias will be evaluated by inspection of the funnel plot which will relate the standard errors of studies to their event rates. If inspection of the funnel plot suggested possibility of publication bias, the pooled prevalence of COVID-19-related OD will be corrected for publication bias by calculation using the trim-and-fill method. Egger’s test will also be performed. Meta-regression will be used to estimate the extent to which measured covariates (sex ratio, subject ethnicity, the mean age of the patients, the proportion of subjects with COVID-19-related OD as the first symptom, and the mean SARS-CoV-2 viral load of relevant clinical specimens) could explain the

variance between the studies. For all tests, p-Value less than 0.05 will be deemed significant. All analyses will be performed using the comprehensive Meta-Analysis version 3 (https://www.meta-analysis.com/index.php) (Biostat; Englewood, NJ, USA). Descriptive statistics will be used for outcomes that are not suitable for meta-analyses.

29. * Analysis of subgroups or subsets.

State any planned investigation of ‘subgroups’. Be clear and specific about which type of study or participant will be included in each group or covariate investigated. State the planned analytic approach.

Subgroup analysis and assessments will be performed according to the following:

[A] Olfactory dysfunction in COVID-19 patients, documented by qualitative assessments (e.g. self-reporting, surveys, questionnaires) only

[B] Olfactory dysfunction in COVID-19 patients, documented by quantitative measurements (e.g. butanol threshold test, smell identification test...)

30. * Type and method of review.

Select the type of review, review method and health area from the lists below.

Type of review

Cost effectiveness
No

Diagnostic
No

Epidemiologic
No

Individual patient data (IPD) meta-analysis
No

Intervention
No

Meta-analysis
Yes

Methodology
No

Narrative synthesis
No

Network meta-analysis
No

Pre-clinical
No

Prevention
No

Prognostic
No

Prospective meta-analysis (PMA)
No

Review of reviews
No

Service delivery
No

Synthesis of qualitative studies
No

Systematic review
Yes

Other
No

Health area of the review

Alcohol/substance misuse/abuse
No

Blood and immune system
No

Cancer
No

Cardiovascular
No

Care of the elderly
No

Child health
No

Complementary therapies
No

COVID-19
Yes

Crime and justice
No

Dental
No

Digestive system
No

Ear, nose and throat
Yes

Education
No

Endocrine and metabolic disorders
No

Eye disorders
No

General interest
No

Genetics
No

Health inequalities/health equity
No

Infections and infestations
No

International development
No

Mental health and behavioural conditions
No

Musculoskeletal
No

Neurological
Yes

Nursing
No

Obstetrics and gynaecology
No

Oral health
No

Palliative care
No

Perioperative care
No

Physiotherapy
No

Pregnancy and childbirth
No

Public health (including social determinants of health)
No

Rehabilitation
No

Respiratory disorders
Yes

Service delivery
No

Skin disorders
No

Social care
No

Surgery
No

Tropical Medicine
No

Urological
No

Wounds, injuries and accidents
No

Violence and abuse
No

31. Language.

Select each language individually to add it to the list below, use the bin icon to remove any added in error.
English

There is not an English language summary

32. * Country.

Select the country in which the review is being carried out. For multi-national collaborations select all the countries involved.

Hong Kong

33. Other registration details.

Name any other organisation where the systematic review title or protocol is registered (e.g. Campbell, or The Joanna Briggs Institute) together with any unique identification number assigned by them. If extracted data will be stored and made available through a repository such as the Systematic Review Data Repository (SRDR), details and a link should be included here. If none, leave blank.

34. Reference and/or URL for published protocol.

If the protocol for this review is published provide details (authors, title and journal details, preferably in Vancouver format)

Add web link to the published protocol.

Or, upload your published protocol here in pdf format. Note that the upload will be publicly accessible.

No I do not make this file publicly available until the review is complete

Please note that the information required in the PROSPERO registration form must be completed in full even if access to a protocol is given.

35. Dissemination plans.

Do you intend to publish the review on completion?

Yes

Give brief details of plans for communicating review findings.?

36. Keywords.

Give words or phrases that best describe the review. Separate keywords with a semicolon or new line. Keywords help PROSPERO users find your review (keywords do not appear in the public record but are included in searches). Be as specific and precise as possible. Avoid acronyms and abbreviations unless these are in wide use.

COVID-19 SARS-CoV-2

Olfactory dysfunction

Anosmia

Smell disturbancesSmell loss

Systematic review

Meta-analysis

37. Details of any existing review of the same topic by the same authors.

If you are registering an update of an existing review give details of the earlier versions and include a full bibliographic reference, if available.

38. * Current review status.

Update review status when the review is completed and when it is published.New registrations must be ongoing.
Please provide anticipated publication date

Review_Ongoing

39. Any additional information.

Provide any other information relevant to the registration of this review.

40. Details of final report/publication(s) or preprints if available.

Leave empty until publication details are available OR you have a link to a preprint. List authors, title and journal details preferably in Vancouver format.

Give the link to the published review or preprint.
